# Why Treat Apogeotropic BPPVs of the Horizontal Canal? About 30 Observations

**DOI:** 10.1155/2011/278383

**Published:** 2011-07-21

**Authors:** Philippe Lorin, Francois Foubert, Marie Debaty

**Affiliations:** ^1^Ear Nose Throat, Vertigo and Vestibular Rehabilitation Clinic Center, 15 rue Gougeard, 72000 Le Mans, France; ^2^Medical Statistic Department, Hospital Center, 194 avenue Rubillard, 72000 Le Mans, France; ^3^Ear Nose Throat, Saint-Pierre Hospital Center, Porte de Hal, 1000 Bruxelles, Belgium

## Abstract

Benign paroxysmal positional vertigo (BPPV), of the horizontal canal, in the apogeotropic form (AHBPPV) was described in 1995. Based on 30 observations of typical AHBPPVs of the horizontal canal, we endeavor to discuss the relevance of physiotherapy. *Material and Method.* Thirty observations of typical apogeotropic BPPVs of the horizontal canal treated with a 360° barbeque rotation on the BPPV side, reviewed in consultation at 1 and 3 weeks and reevaluated the following year. *Results.* Our cohort of 30 patients had an average age of 58.6 years. The apogeotropic BPPVs of the horizontal canal, which can be transformed into BPPVs of the posterior canal or into geotropic-type BPPVs of the horizontal canal do not recover more quickly. Patients who follow the positional advice do not recover more quickly than those who do not (*P* = 0.152). The 15 patients treated on average 13.73 days after the onset of the disease did not recover more quickly after the start of therapeutic treatment than those treated later (*P* = 0.032). *Conclusion.* Here, we demonstrate that the direction of rotation during the maneuvers is of no importance for the results. We show that transformability is not a guarantee of rapid recovery and that the therapist's effectiveness is limited when it comes to the short-term results.

## 1. Introduction

Benign paroxysmal positional vertigo (BPPV) of the horizontal canal was described in 1985 [1] as geotropic form (downbeating nystagmus, geotropic BPPV of the horizontal canal, GHBPPV). The apogeotropic form has been described more recently in 1995 [2]. This form, apogeotropic BPPV of the horizontal canal (AHBPPV), is characterized by an upbeating horizontal nystagmus provoked by a right or left lateral decubitus.

It accounts for between 16 and 26% of BPPVs of the horizontal canal [3], that is, approximately 2 to 4% of BPPVs where an impact on the posterior canal is dominant, an incidence which is slightly higher than that of the BPPVS of the anterior canal [4]. For 15 years now and since the first description of BALOH, therapeutic solutions have been discussed and must be validated. 

Based on 30 observations of typical apogeotropic BPPVs of the horizontal canal, we endeavor here to discuss the relevance of physiotherapy and what it offers patients in terms of comfort and symptomatic relief.

## 2. Material and Method

### 2.1. The Patients

Between 2006 and 2007, 30 patients came to the clinic for exploration and vestibular rehabilitation for treatment of a typical AHBPPV, without central cause possible (MRI and neurologic examination normal).

### 2.2. The Method

#### 2.2.1. The Treatment

Each patient underwent a 360° barbeque rotation maneuver of the affected side.

To determine which side of the AHBPPV was affected we used the following arguments  in order: (1) the side where the nystagmus was the weakest, (2) then the side in which vertigos were the weakest, (3) and lastly the way the nystagmus went to when the head was bent forward (bow and lean test).

The patients were reviewed during a systematic control at 1 and 3 weeks. A 360° barbeque rotation on the affected side was undertaken for the apogeotropic BPPVs of the horizontal canal.

When, during a session, we transformed the AHBPPV, we immediately performed the adapted maneuver: 270° LEMPERT's Barbeque roll maneuver on the side of the healthy ear on a GHBPPV and SEMONT's liberatory maneuver on a posterior canal BPPV (PBPPV).

Eight pieces of prophylactic advice were given after each maneuver. 

In the case of an AHBPPV, these positional pieces of advice consisted of  (1) sleep on the BPPV side, (2) put the night stand on the side of the BPPV, (3) do not make housework with the head tilted back, (4) or bent forward, (5) do not practice makeshift job with the head tilted back, (6) or bent forward, (7) do not work in garden with the head tilted back, (8) or bent forward.

#### 2.2.2. The Retrospective Information from the Clinical File

Data were compiled from the clinical file on sex, age, time to therapeutic treatment, the side affected, transformability (into BPPVs of the posterior canal or geotropic BPPVs of the horizontal canal), the time to symptomatic recovery, and the number of maneuvers or consultations needed to obtain symptomatic recovery. The symptomatic or clinical recovery was obtained with disappearance of positional vertigo. The videoscopic recovery was obtained with disappearance of videoscopic positional nystagmus.

#### 2.2.3. Retrospective Information on the Control Consultation

Thirty patients were reviewed in consultation in 2008 and we undertook the following.

1. An evaluation of the residual symptoms using the Vertigo Symptom Scale (VSS) [5] and the Dizziness Handicap Inventory (DHI) [6]. VSS and DHI are expressed as a total out of 100 points. The symptomatic score of our patients is expressed as a total out of 200 points (sum of VSS and DHI).

(2) An evaluation lasting 10 seconds of residual nystagmus in videonystagmoscopy in the Head Shaking Test, in anteflexion, in right HALLPIKE, and left HALLPIKE, in right lateral decubitus and left lateral decubitus. The score was recorded as 1 if there was nystagmus, as 0 if there was none. The total score of each patient was evaluated in respect of 6 points.

(3) An evaluation of the following prophylactic advice given at the end of each consultation. This following of the positional advice was evaluated for 8 items using the values 1 (always), 2 (almost always), 3 (almost never), 4 (never). The total number of points obtained for each of these items enabled us to calculate a positional risk score out of 32 points.

### 2.3. Statistics

We used the SPSS software. For the comparisons of quantitative values we drew on the Fischer test, and a *P* < 0.05 was used as the test for a significant hypothesis. We then compared the averages between the two groups of 15 on both sides of the median. The correlations were evaluated using the Pearson score.

## 3. Results

Table 1 gives the diagnosis elements, the delays, the transormability and the evolution. This cohort of 30 patients had a gender ratio of 11 men to 19 women, 36.66%.

The average age was 58.6 (standard deviation 15.22, median 63, extremes 24 to 82 years of age). The positional vertigos on the day of the first consultation had been developing for 11.6 days on average (standard deviation 10.90, median 9, extremes 1 to 40 days). In 19 cases (63.33%), we determined the affected side thanks to the side in which the nystagmus in lateral decubitus (NLD) was the weakest. We used the side wherein vertigo was the weakest in 5 cases (16.66%, when right NLD = left NLD). Lastly, in 6 cases (20%) we delineated it thanks to the nystagmus caused by the head bent forward (bow and lean test, when right NLD = left NLD and right vertigo = left vertigo).

Fifteen patients had a right apogeotropic, 15 a left apogeotropic BPPV of the horizontal canal.

About the transformability at first consultation (C1 on Table 1), after the first maneuver (M1 on Table 1), 5 AHBPPV were transformed in GHBPPV. 3 of them remained on the second consultation, the 2 others became a PBPPV and a AHBPPV. 

Regarding transformability and time to recovery at the second consultation (C2 on Table 1), we observed in our cohort during the first control consultation after 8.6 days on average: 15 patients who had recovered in terms of symptoms, but only 8 who had recovered from the angle of videoscopy (no positional nystagmus). In the case of the 22 others: 11 patients with a transformed BPPV (4 into PBPPV and 7 into GHBVVP) and 11 AHBPPVs of the horizontal canal. 

Regarding transformability and time to recovery at the third consultation (C3 on Table 1, second control consultation) after 14.8 days on average we obtained for our 22 cases: 18 symptomatic recoveries and 14 videoscopic recoveries. For the 8 other patients with positive videoscopy we obtained 3 transformed BPPVs (1 BPPV of the posterior canal and 2 geotropic BPPVs of the horizontal canal) and 5 apogeotropic BPPVs of the horizontal canal.

Finally, the number of maneuvers and the number of consultations necessary to obtain videoscopic recovery are linked, but they also depart from each other. The average number of consultation is 2 and the average number of maneuvers is 2.2 for all our cases. If we only consider the 28 patients clinically cured on the third consultation, the average number of consultations is 1.92 and the average number of maneuvers is 2.10.

Table 2 summarizes our statistical study and the Fischer test. We examined the impact of the six factors on symptomatic and videoscopic recovery some time after the initial episode.

### 3.1. Influence of the Factor “Age”

There is no significant difference regarding the distance in terms of time from the initial episode between the young and older groups of patients. Their symptomatic and videoscopic scores are similar and for these two elements the small *P* values are not significant. However, we do note a significant difference between the number of consultations needed to relieve a young patient (1.6) and that needed to relieve older patients (2.4, *P* = 0.002). This can perhaps be explained by the results of the following column since it seems that the young patients are treated more quickly (10.4 days) whereas the older patients are only treated after 12.8 days (*P* = 0.05).

### 3.2. Influence of the Factor “Transformability”

We did not observe any significant differences immediately (number of consultations or acts) or at a later stage (VSS, DHI, VNS) between the group of apogeotropic BPPVs of the horizontal canal transformable (into BPPVs of the posterior canal or geotropic BPPVs of the horizontal canal) and the nontransformable group of apogeotropic BPPVs of the horizontal canal. Our two subgroups were similar and comparable in terms of age, time to consultation, and time to recovery. They had both followed the positional advice in an identical manner. All the small *P* values were greater than 0.1 for this criterion.

### 3.3. Influence of the Factor “Positional Risk”

Whether the patients completely followed the positional advice or not, their VSS, DHI, and VSS scores were unchanged some time after the initial episode. Our two sub-groups were comparable; all the small *P*-values were greater than 0.152 for this criterion.

### 3.4. Influence of the Factor “Scarring Time”

The time between the first maneuver and the control consultation at a later date does not influence the VSS, DHI, or VNS results either. The two sub-groups were comparable from the point of view of the other criteria and the small *P*-values were all greater than 0.193.

### 3.5. Influence of the Factor “Number of Consultations”

The number of consultation is linked with the number of maneuvers. The study of this compounding factor reveals that the patients for whom clinical recovery was secured the most quickly are the ones who had the best prognosis of videoscopic recovery some time after the episode (*P* = 0.04). The clinical or symptomatic recovery is link with the videoscopic recovery. But this does not have any impact on the symptomatic results or the patient's impression 10.76 months after the episode (*P* = 0.81). A videoscopic or clinical regaining of a patient immediately after the maneuvers does not mean that its symptomatic scores will be good.

### 3.6. Influence of the Factor “Time to Treatment”

The patients treated 3.73 days on average after the onset of the disease recovered more quickly than those treated 19.47 days after the onset of the disease (*P* = 0.032). The number of consultations may be linked to this result. Nonetheless at a later stage, the two groups are doing equally well or poorly from the point of view of VSS, DHI, and VNS with small *P*-values greater than 0.5. 

Our study concerning statistics and correlation with the Pearson test does not reveal any interesting results.

There is no correlation between the symptomatic results and the patients' age (*r* = 0.129), no correlation between the symptomatic results and the following of positional advice (*r* = −0.148), and no correlation between the symptomatic results and videoscopic recovery (*r* = 0.110).

Nor is there any correlation between the videoscopic results and the patients' age (*r* = 0.189), the videoscopic results and the following of positional advice (*r* = −0.373), and finally no correlation between age and the following of positional advice (*r* = −0.052).

## 4. Discussion

Etiologic diagnosis and lateralization of AHBPPV are difficult, still contentious, and disputed. The hypothesis of a cupulolithiasis [7] or a canalolithiasis located in the ductal side next to the cupule can be retained [8, 9]. According to some authors, sometimes a central etiology can be mentioned [10, 11]. In this study we only took in account the AHBPPV for which imaging and neurologic examination were normal. For an AHVPPB, the healthy side is the side where the apogeotropic nystagmus is the strongest [2, 9, 12], but it is also the side in which the vertigo felt by the patient is the most intense. It seems to these authors that the bow and lean test [13–15] is an important element but it needs a videoscopy. The nystagmus beats on the healthy side when the head is bent forward. It is reversed when the head is tilted back. 

For this study we only selected AHVPPB for which arguments of lateralization matched. We aimed at corroborate or invalidate the efficiency of the assigned therapy. We preferred to exclude some AHVPPBs for which we were doubtful about the side affected. For that, we observed the diagnostic recommendations of the literature. Thus, when the apogeotropic nystagmus was too intense to enable a differentiation (11 cases), we asked the patient to tell us the side where vertigo was the strongest and in 5 cases this argument enabled us to lateralize the AHBPPV. In the 6 remaining cases we lateralized the BPPV thanks to the bow and lean test. 

The treatment of geotropic BPPV of the horizontal canal was described before that of apogeotropic BPPV of the horizontal canal. The first articles dealing with this subject mention the possible effectiveness of therapeutic rotations in the horizontal canal plan [7, 16] for geotropic BPPVs of the horizontal canal. Unfortunately, these first references do not clearly indicate the rotation direction in these maneuvers. The concept of rotation in the opposite sense to the geotropic BPPV of the horizontal canal with at least 270° has become the accepted norm over the course of time [17] as well as postmaneuver recommendations along with, in some cases, extended decubitus on the side opposite the geotropic BPPV of the horizontal canal. The literature on this subject concerning apogeotropic BPPVs of the horizontal canal (scale of rotation, direction of rotation, and postmaneuver advice) is less clear and much debated. It is accepted that the transformation of an apogeotropic BPPV of the horizontal canal into a geotropic BPPV of the horizontal canal results from the displacement of the free-floating otoliths in the anterior section of the horizontal canal towards the posterior section [13]. Furthermore, this is theoretically, anatomically, and clinically conceivable. For some authors this transformation of an apogeotropic BPPV of the horizontal canal into a geotropic BPPV of the horizontal canal is a necessary preliminary. They admit that, as geotropic BPPVs of the horizontal canal are easier to treat than apogeotropic BPPVs of the horizontal canal, transformable apogeotropic BPPVs of the horizontal canal will be easier to control [16, 18]. These different authors agree and suggest for apogeotropic BVVPs of the horizontal canal an approach maneuver that contradicts the one generally accepted as being effective in the treatment of geotropic BPPVs of the horizontal canal. Therapeutic rotation can be undertaken on the side opposite to the apogeotropic BPPV of the horizontal canal (anticlockwise/unaffected side for the ones on the right AHBPPV and clockwise/unaffected side for the ones on the left AHVPPB) with a scale of 270° at least and/or in some cases recommendations of extended sleep on the affected side. It is also possible to practice a repositioning maneuver to transform AHBPPV in GHBPPV [18, 19].

Our study has shown that the rotation direction in contradiction to the normal physiopathological direction and to the one recommended in these articles [7, 16] could be effective. Consequently, our study challenges the therapeutic rotation direction accepted for these apogeotropic BPPVs of the horizontal canal. It seems that this direction does not influence the results. In contrast to our expectations and the literature, we have shown that irrespective of whether a barbeque maneuver or a Guffoni maneuver is involved [18, 19], transformability did not in any way predict the sensitivity of apogeotropic BPPVs of the horizontal canal to these maneuvers, that this transformability did not permit more rapid control of apogeotropic BPPVs of the horizontal canal and that the videoscopic and symptomatic results of these apogeotropic BPPVs of the horizontal canal, whether transformable or not, were identical. Whether the AHBPPVs can be transformed into GHBPPVs or not, whether treated with an affected side barbeque maneuver or not, the results are similar, maybe with better results to the unaffected ear rotation. FIFE [7] with 6 AHBPPV, after one week and an affected ear barbeque 360° maneuver obtained 4 recoveries (66%). GUFONI [18] with 6 AHBPPV, after one week and an unaffected ear barbeque 270° maneuver obtain 6 recoveries (100%) and 4 transformations (66%). In our study with 30 AHBPPV after 4 weeks and an affected ear barbeque 360°maneuver, we obtained 22 recoveries (73%) and 13 transformations (43%).

Given the physiopathological hypotheses involving otolithic migration indicated in the apogeotropic BPPVs of the horizontal canal and given the accepted knowledge concerning BPPVs of the posterior canal and geotropic BPPVs of the horizontal canal, we rightly believe that a shift towards spontaneous healing of apogeotropic BPPVs of the horizontal canal is possible [8]. Furthermore, this has already been mentioned in conjunction with small series [17]. The simple fact of sleeping on the affected side over a prolonged period can lead to recovery [17]. We believe it would be interesting to conduct research within this framework into the potential noninfluence of the therapist if external factors could prove to be effective. 

We have shown here that as the patient's age increases, the number of acts needed to achieve recovery rose significantly (*P* = 0.002). We have shown also than an elevated number of consultations (or acts or maneuvers) increase videoscopic score after 10.76 months. The more the AHBPPV needs maneuvers the more its videoscopy at a distance is distorted. We have demonstrated that neither the scarring time nor the following of positional advice modified the symptomatic results (*P* = 0.428 and *P* = 0.792, resp.). Thus, in the case of apogeotropic BPPVs of the horizontal canal, rapid consultation (within a few days) involving videonystagmoscopy by a therapist significantly reduced the time required for short-term recovery particularly if the patient is young. Nonetheless, a few months later, the symptomatic and videoscopic status of all patients will be the same. It can, therefore, be stated that this treatment gives symptomatic comfort for a few days (3.73 days low average—19.47 days high average) to patients who will seemingly recover spontaneously.

In this study we also demonstrate that there is no correlation between the criteria for videoscopic and symptomatic recovery. This opens the door to a new concept for defining recovery from apogeotropic BPPVs of the horizontal canal. When it comes to assessing recovery, should we—as therapists—turn our attention to nystagmic criteria like we do for diagnosis [20]? Or should we focus on symptomatic criteria [5, 6], tools that have been validated in the past and seem to be more suited? We likewise demonstrate that there is no longer any correlation between the following of positional advice and symptomatic or videoscopic results. We could have imagined that age would influence these results but it does not.

## 5. Conclusion

Fifteen years after the first description of an apogeotropic BPPV of the horizontal canal, the therapeutic method is still a subject of debate. Based on 30 typical observations, we have demonstrated that the direction of rotation during the maneuvers is of no importance, that transformability was not a measure of positive results in the long term and that the effectiveness of the therapist regarding the short-term results was limited in our experience. Some external predetermined factors like age and time to consultation seem to be important. There does not seem to be any link between the symptomatic results, the videoscopic observations or the following of positional advice. Our next study will endeavor to compare our therapeutic results with complete abstention. The hypothesis of multiple etiologies, including some which fail to respond to physiotherapy in the treatment of apogeotropic BPPVs of the horizontal canal, should be mentioned.

## Figures and Tables

**Table 1:** The elements which enabled the authors to undertake the diagnosis, the delays, the transformability and the evolution.

Case	Sex	Age	Duration before C1	Dominant side nystagmus	Dominant side vertigo	Bow test nystagmus	Side of BPPV	VNS type of BPPV at C1	VNS type of BPPV after M	Time between C1 and C2	Vertigo at C1	VNS type of BPPV at C2	VNS type of BPPV after M	Time between C2 and C3	Vertigo at C3	VNS type of BPPV at C3	VNS type of BPPV after M	Total of M	Total of C
			(days)							(days)				(days)					
1	M	46	13	R > L	R > L	0	L	APO	APO	20	No							1	1
2	M	47	5	L = R	L = R	R	L	APO	APO	6	No							1	1
3	M	67	5	R > L	R > L	R	L	APO	APO	5	No							1	1
4	F	54	1	L > R	L > R	L	R	APO	APO	3	No							1	1
5	F	53	21	L > R	L = R	L	R	APO	APO	19	No							1	1
6	F	24	1	L > R	L > R	L	R	APO	APO	5	No							1	1
7	M	29	28	L > R	L > R	L	R	APO	APO	20	No							1	1
8	M	54	2	R > L	R = L	R	L	APO	APO	4	No							1	1
9	F	64	2	L = R	L = R	R	L	APO	APO	7	No	APO	APO	37	No			2	2
10	F	66	8	L > R	L > R	0	R	APO	GEO	8	No	APO	APO	14	No			3	2
11	F	74	4	L > R	L > R	0	R	APO	APO	4	Yes	APO	GEO	10	No			3	2
12	F	49	10	R > L	R > L	R	L	APO	APO	10	Yes	APO	APO	31	No			2	2
13	F	64	12	L = R	R > L	R	L	APO	APO	4	Yes	APO	APO	8	No			2	2
14	F	80	2	L = R	R > L	R	L	APO	GEO	4	No	GEO	GEO	42	No			3	2
15	F	41	9	L > R	L = R	0	R	APO	APO	5	Yes	GEO	GEO	21	No			2	2
16	M	47	2	R > L	R > L	0	L	APO	APO	10	Yes	GEO	GEO	14	No			2	2
17	F	63	26	L > R	L = R	0	R	APO	APO	8	Yes	GEO	GEO	30	No			2	2
18	F	43	3	L = R	L = R	L	R	APO	APO	4	Yes	GEO	GEO	20	No			2	2
19	M	47	10	L > R	L > R	L	R	APO	GEO	5	No	POS	POS	21	No			3	2
20	F	80	40	L = R	L = R	R	L	APO	APO	8	No	POS	POS	15	No			2	2
21	F	82	15	L = R	L = R	L	R	APO	APO	6	No	POS	POS	15	No			2	2
22	F	50	13	L > R	L = R	L	R	APO	APO	6	No	POS	POS	12	No			2	2
23	F	77	11	R > L	R > L	R	L	APO	APO	17	Yes	APO	APO	47	No	APO	APO	3	3
24	M	63	1	L = R	L > R	0	R	APO	APO	2	Yes	APO	APO	6	No	APO	APO	3	3
25	F	75	8	R > L	R > L	R	L	APO	APO	16	Yes	APO	APO	30	No	APO	APO	3	3
26	M	75	40	L = R	L > R	0	R	APO	APO	6	Yes	APO	APO	13	No	APO	APO	3	3
27	F	64	26	L = R	R > L	R	L	APO	APO	20	Yes	APO	APO	49	No	APO	APO	3	3
28	F	65	3	L = R	L = R	R	L	APO	GEO	9	No	GEO	GEO	44	No	GEO	GEO	4	3
29	M	72	15	R > L	L = R	R	L	APO	GEO	11	Yes	GEO	GEO	15	Yes	GEO	GEO	4	3
30	M	43	12	L > R	L > R	L	R	APO	APO	6	Yes	APO	APO	21	Yes	POS	POS	3	3

**Table 2:** Summarizes our statistical study and the Fischer test.

	Average age	Average positional risk	Average scarring time	Average number of consultations before recovery	Average time to consultation	Average VSS and DHI scores	Average videoscopic score
Young subjects	46	15.08	10.27	1.6	10.4	42.67	3.6
Older subjects	71.2	16.02	11.27	2.4	12.8	43.87	4
Small *P*	**0**	0.307	0.76	**0.002**	**0.05**	0.918	0.892
Transformable AHBPPVs	56	15.42	8.25	1.88	9.81	40.13	3.52
Non-transformable AHBPPVs	61.2	15.68	13.29	2.12	13.39	46.41	4.09
Small *P*	0.375	0.307	0.101	0.333	0.346	0.563	0.547
Low positional risk	59.4	13.44	8.4	1.87	10.53	44.8	4.53
High positional risk	57.8	17.66	13.13	2.13	12.67	41.73	3.07
Small *P*	0.779	**0**	0.152	0.334	0.601	0.792	0.701
Short scarring time	56.2	14.8	3.87	1.93	11.3	38.67	4.26
Long scarring time	61	16.3	17.67	2.07	11.9	47.87	3.34
Small *P*	0.397	0.193	**0**	0.631	0.819	0.428	0.482
Low number of consultation	57.2	15	9.2	1.53	10.4	44.67	2.8
High number of consultation	60	16.1	12.33	2.47	12.8	41.87	4.8
Small *P*	0.623	0.307	0.348	**0**	0.556	0.81	**0.04**
Short time to consultation	57.6	15.73	11	1.57	3.73	39.73	4.27
Long time to consultation	59.6	15.37	10.53	2.43	19.47	46.8	3.33
Small *P*	0.326	0.636	0.89	**0.032**	**0**	0.543	0.603
